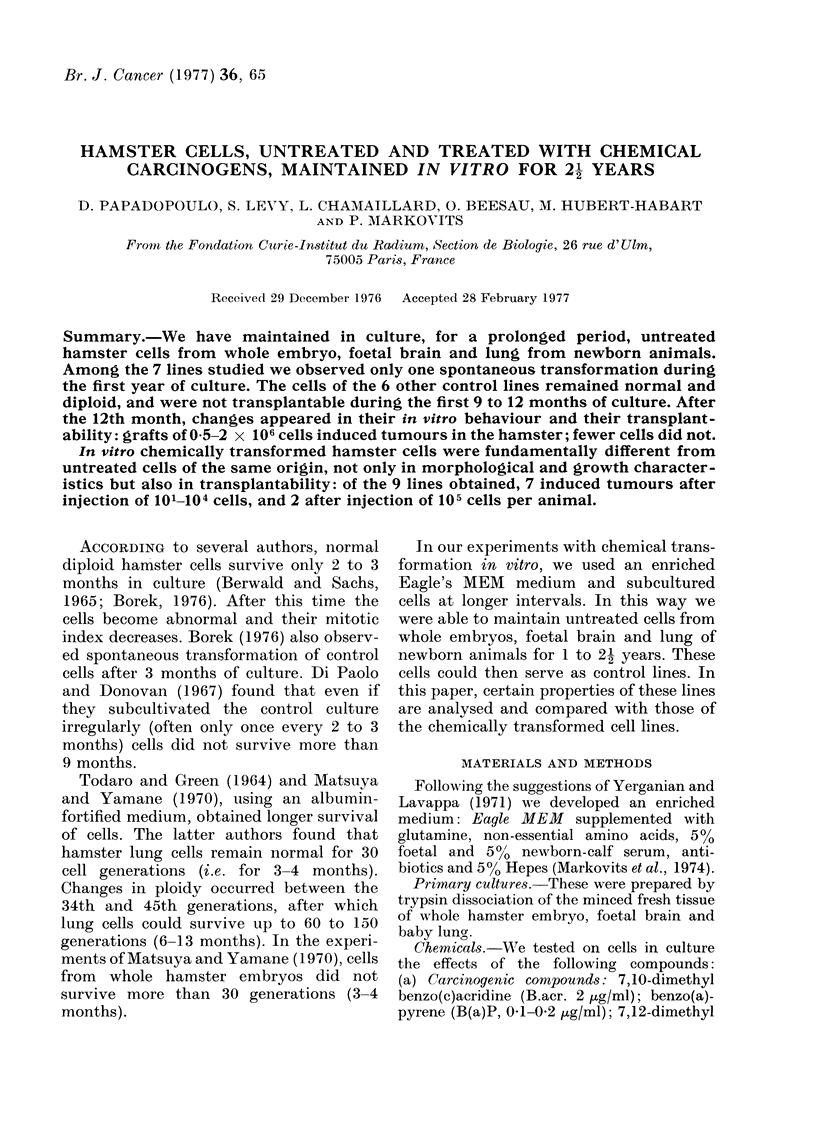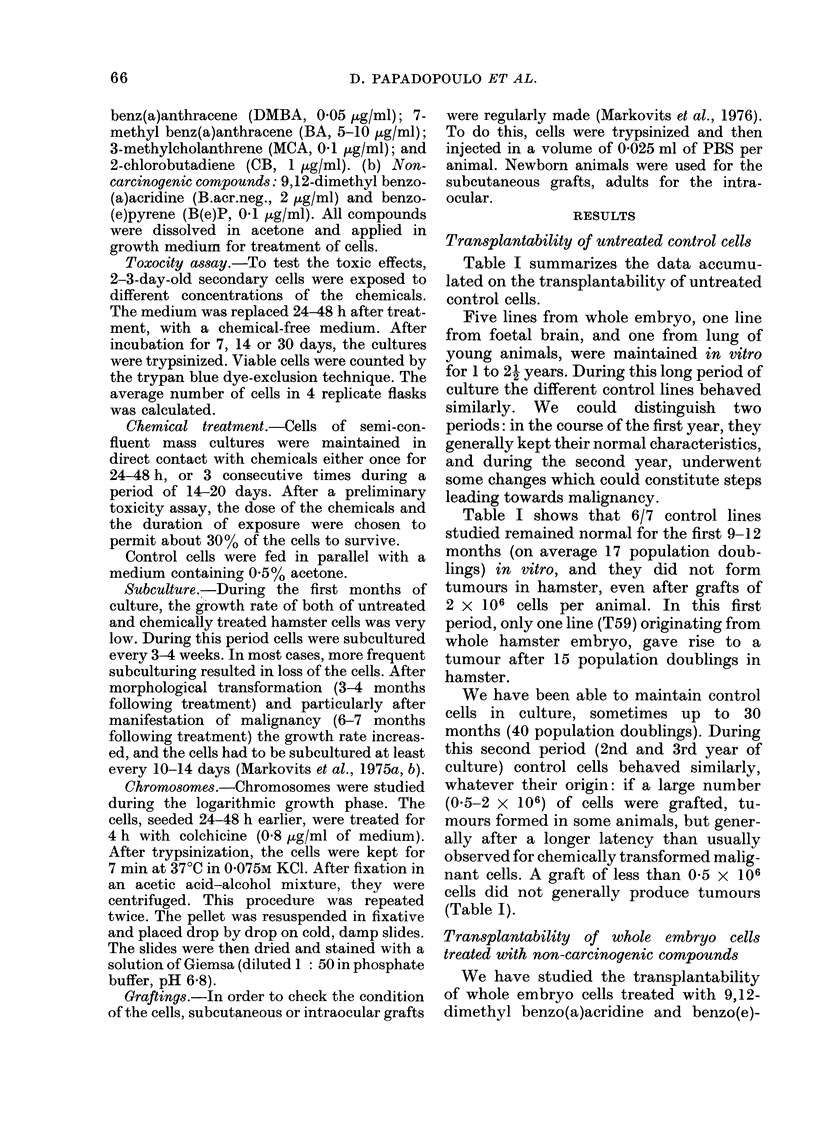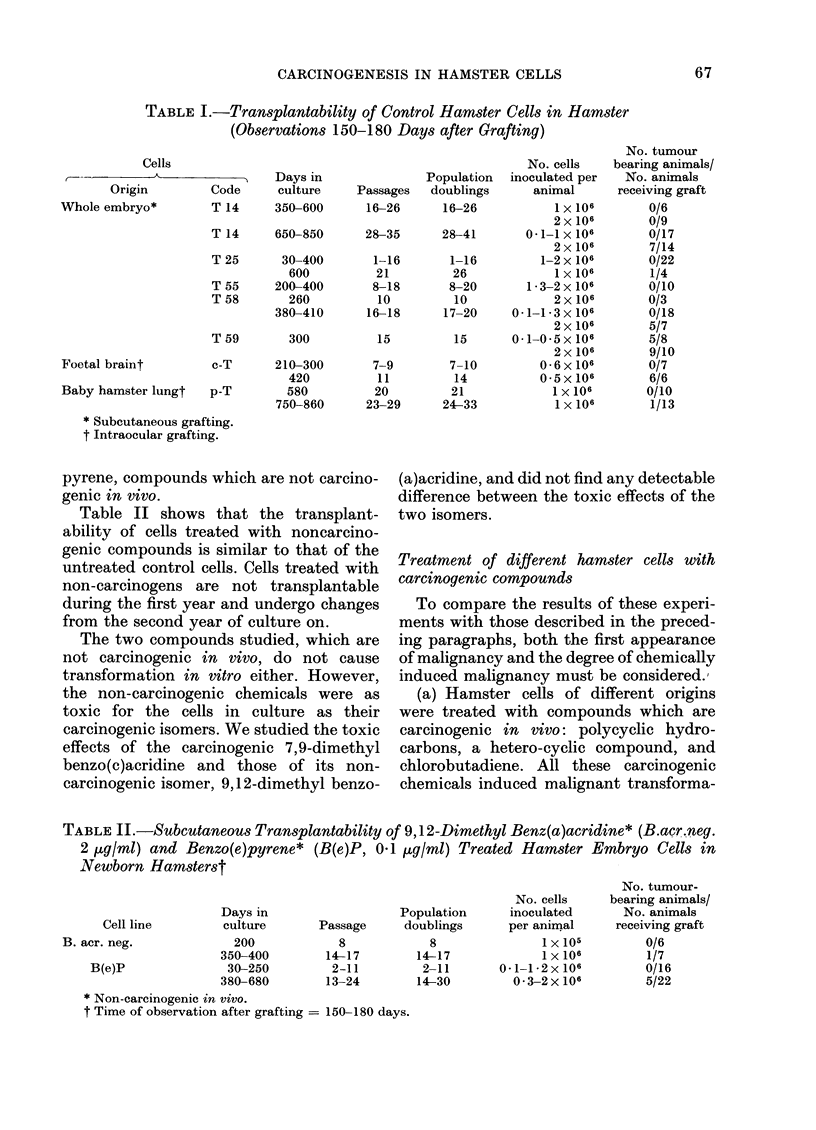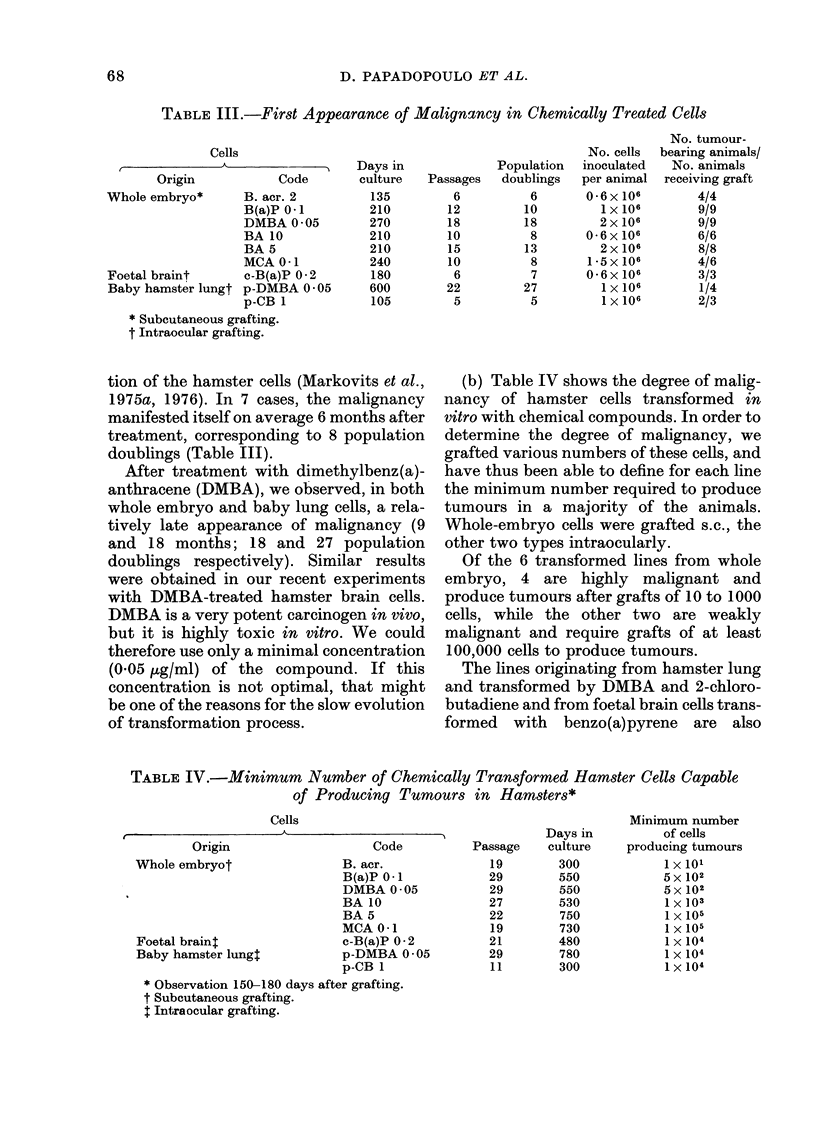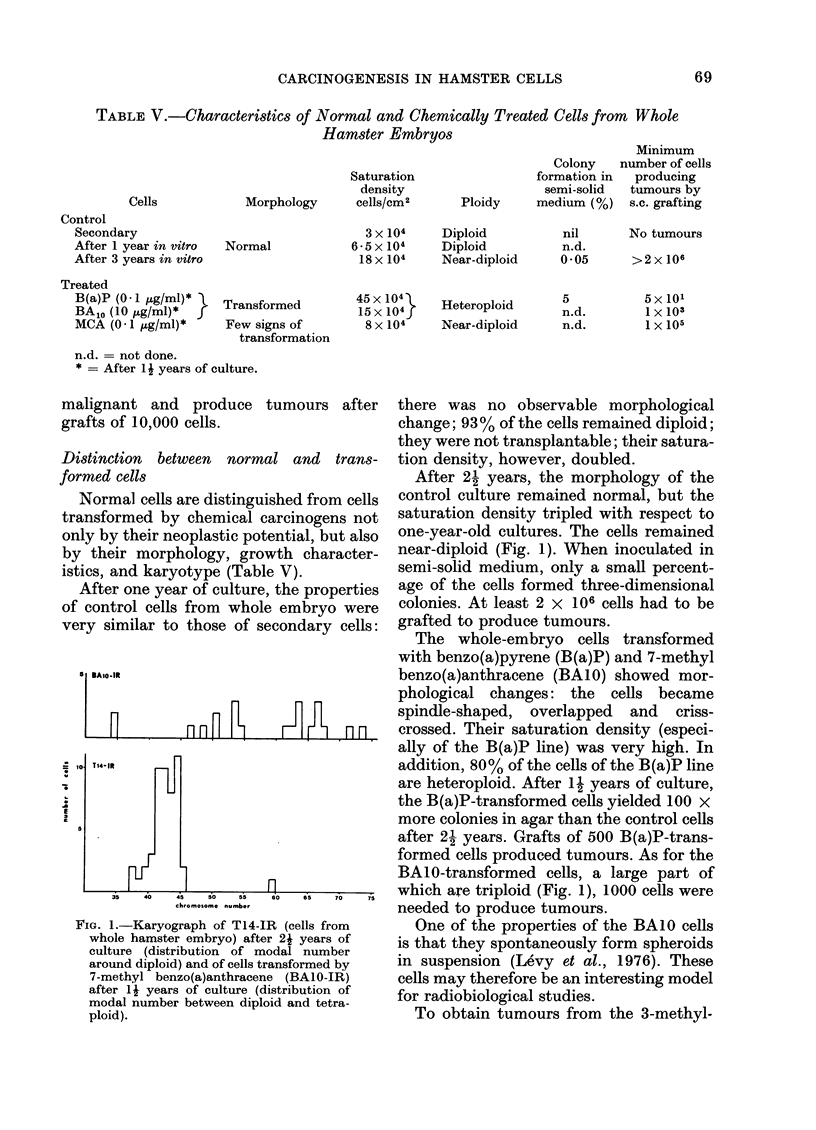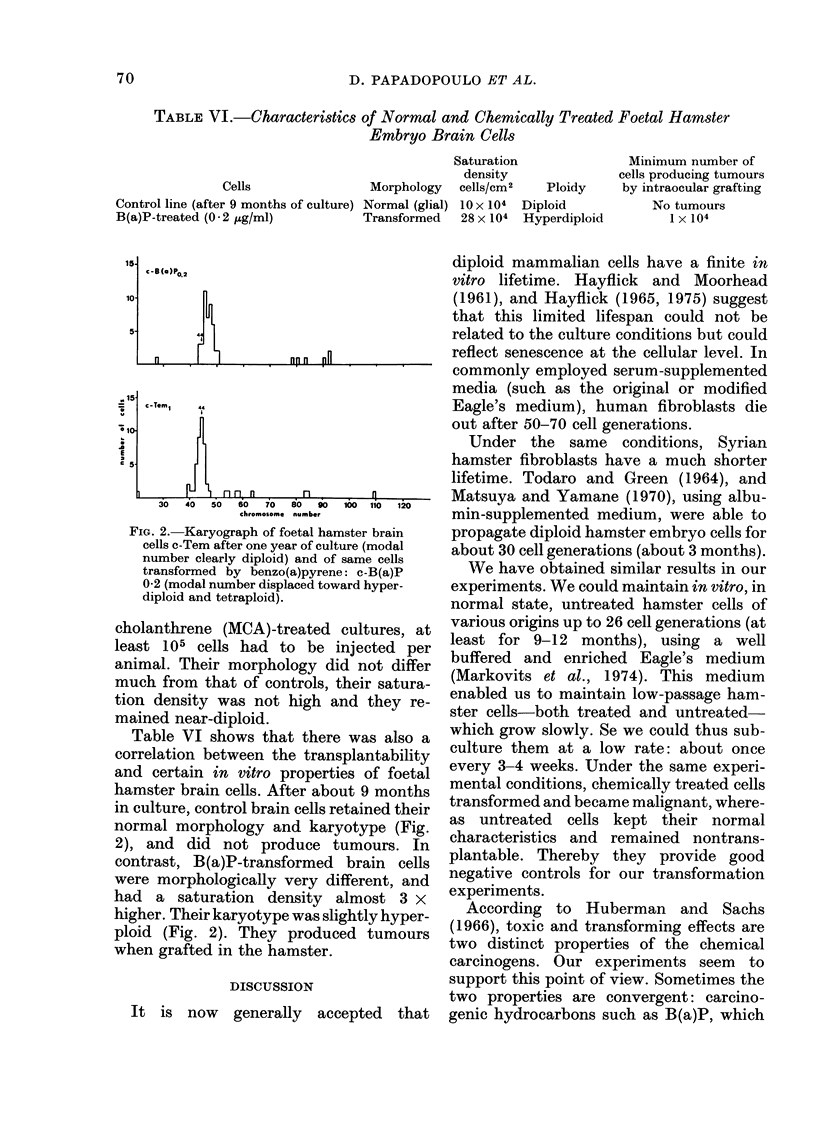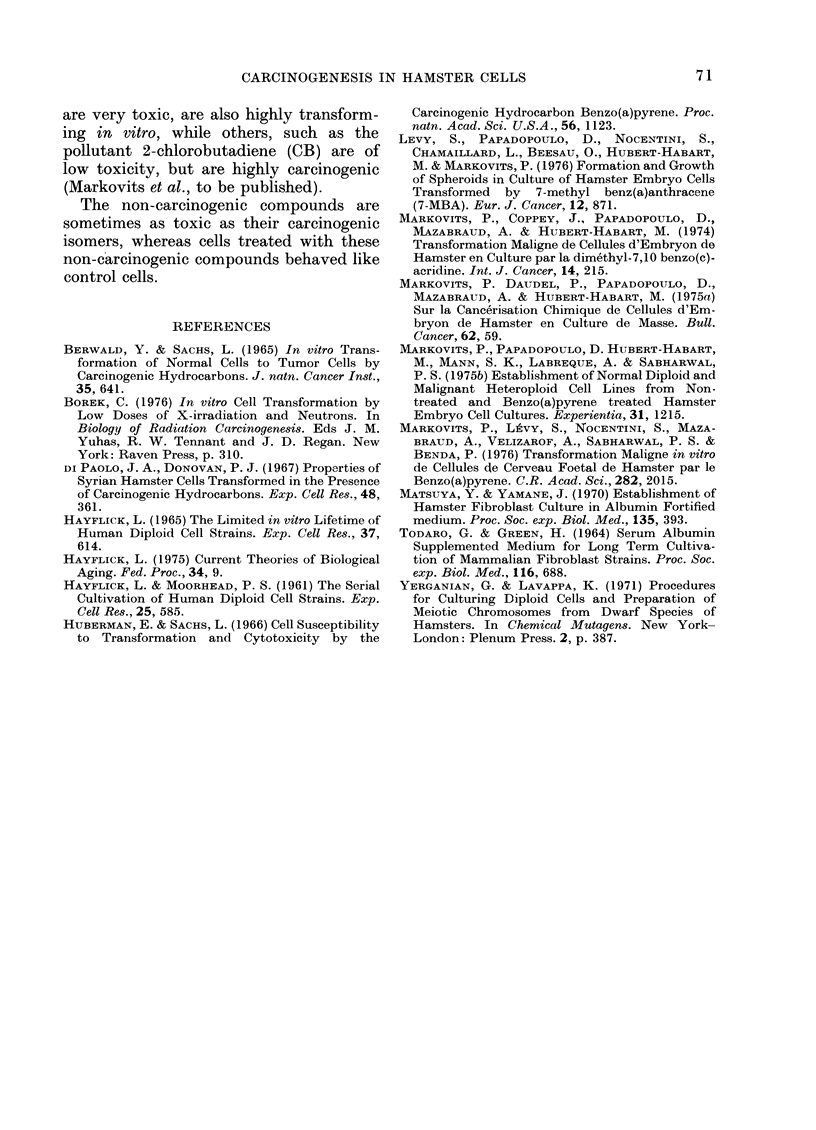# Hamster cells, untreated and treated with chemical carcinogens, maintained in vitro for 2 1/2 years.

**DOI:** 10.1038/bjc.1977.155

**Published:** 1977-07

**Authors:** D. Papadopoulo, S. Levy, L. Chamaillard, O. Beesau, M. Hubert-Harbart, P. Markovits

## Abstract

We have maintained in culture, for a prolonged period, untreated hamster cells from whole embryo, foetal brain and lung from newborn animals. Among the 7 lines studied we observed only one spontaneous transformation during the first year of culture. The cells of the 6 other control lines remained normal and diploid, and were not transplantable during the first 9 to 12 months of culture. After the 12th month, changes appeared in their in vitro behaviour and their transplantability: grafts of 0-5-2 X 10(6) cells induced tumours in the hamster; fewer cells did not. In vitro chemically transformed hamster cells were fundamentally different from untreated cells of the same origin, not only in morphological and growth characteristics but also in transplantability; of the 9 lines obtained, 7 induced tumours after injection of 10(1)-10(4) cells, and 2 after injection of 10(5) cells per animal.


*Br. J. Cancer* (1977) **36**, 65

### HAMSTER CELLS, UNTREATED AND TREATED WITH CHEMICAL CARCINOGENS, MAINTAINED *IN VITRO* FOR 2½ YEARS

D. PAPADOPOULO, S. LEVY, L. CHAMAILLARD, O. BEESAU, M. HUBERT-HABART AND P. MARKOVITS

*From the Fondation Curie-Institut du Radium, Section de Biologie,* 26 *rue d'Ulm,* 75005 *Paris, France*

Received 29 December 1976 Accepted 28 February 1977

**Summary.—We have maintained in culture, for a prolonged period, untreated hamster cells from whole embryo, foetal brain and lung from newborn animals. Among the 7 lines studied we observed only one spontaneous transformation during the first year of culture. The cells of the 6 other control lines remained normal and diploid, and were not transplantable during the first 9 to 12 months of culture. After the 12th month, changes appeared in their *in vitro* behaviour and their transplantability: grafts of 0·5–2 × $10^6$ cells induced tumours in the hamster; fewer cells did not.**

***In vitro* chemically transformed hamster cells were fundamentally different from untreated cells of the same origin, not only in morphological and growth characteristics but also in transplantability: of the 9 lines obtained, 7 induced tumours after injection of $10^1$–$10^4$ cells, and 2 after injection of $10^5$ cells per animal.**

ACCORDING to several authors, normal diploid hamster cells survive only 2 to 3 months in culture (Berwald and Sachs, 1965; Borek, 1976). After this time the cells become abnormal and their mitotic index decreases. Borek (1976) also observed spontaneous transformation of control cells after 3 months of culture. Di Paolo and Donovan (1967) found that even if they subcultivated the control culture irregularly (often only once every 2 to 3 months) cells did not survive more than 9 months.

Todaro and Green (1964) and Matsuya and Yamane (1970), using an albumin-fortified medium, obtained longer survival of cells. The latter authors found that hamster lung cells remain normal for 30 cell generations (*i.e.* for 3–4 months). Changes in ploidy occurred between the 34th and 45th generations, after which lung cells could survive up to 60 to 150 generations (6–13 months). In the experiments of Matsuya and Yamane (1970), cells from whole hamster embryos did not survive more than 30 generations (3–4 months).

In our experiments with chemical transformation *in vitro*, we used an enriched Eagle's MEM medium and subcultured cells at longer intervals. In this way we were able to maintain untreated cells from whole embryos, foetal brain and lung of newborn animals for 1 to 2½ years. These cells could then serve as control lines. In this paper, certain properties of these lines are analysed and compared with those of the chemically transformed cell lines.

### MATERIALS AND METHODS

Following the suggestions of Yerganian and Lavappa (1971) we developed an enriched medium: *Eagle MEM* supplemented with glutamine, non-essential amino acids, 5% foetal and 5% newborn-calf serum, antibiotics and 5% Hepes (Markovits *et al.*, 1974).

*Primary cultures.*—These were prepared by trypsin dissociation of the minced fresh tissue of whole hamster embryo, foetal brain and baby lung.

*Chemicals.*—We tested on cells in culture the effects of the following compounds: (a) *Carcinogenic compounds:* 7,10-dimethyl benzo(c)acridine (B.acr. 2 μg/ml); benzo(a)-pyrene (B(a)P, 0·1–0·2 μg/ml); 7,12-dimethyl benz(a)anthracene (DMBA, 0·05 μg/ml); 7-methyl benz(a)anthracene (BA, 5–10 μg/ml); 3-methylcholanthrene (MCA, 0·1 μg/ml); and 2-chlorobutadiene (CB, 1 μg/ml). (b) *Non-carcinogenic compounds:* 9,12-dimethyl benzo-(a)acridine (B.acr.neg., 2 μg/ml) and benzo-(e)pyrene (B(e)P, 0·1 μg/ml). All compounds were dissolved in acetone and applied in growth medium for treatment of cells.

*Toxocity assay.*—To test the toxic effects, 2–3-day-old secondary cells were exposed to different concentrations of the chemicals. The medium was replaced 24–48 h after treatment, with a chemical-free medium. After incubation for 7, 14 or 30 days, the cultures were trypsinized. Viable cells were counted by the trypan blue dye-exclusion technique. The average number of cells in 4 replicate flasks was calculated.

*Chemical treatment.*—Cells of semi-confluent mass cultures were maintained in direct contact with chemicals either once for 24–48 h, or 3 consecutive times during a period of 14–20 days. After a preliminary toxicity assay, the dose of the chemicals and the duration of exposure were chosen to permit about 30% of the cells to survive.

Control cells were fed in parallel with a medium containing 0·5% acetone.

*Subculture.*—During the first months of culture, the growth rate of both of untreated and chemically treated hamster cells was very low. During this period cells were subcultured every 3–4 weeks. In most cases, more frequent subculturing resulted in loss of the cells. After morphological transformation (3–4 months following treatment) and particularly after manifestation of malignancy (6–7 months following treatment) the growth rate increased, and the cells had to be subcultured at least every 10–14 days (Markovits *et al.*, 1975*a*, *b*).

*Chromosomes.*—Chromosomes were studied during the logarithmic growth phase. The cells, seeded 24–48 h earlier, were treated for 4 h with colchicine (0·8 μg/ml of medium). After trypsinization, the cells were kept for 7 min at 37°C in 0·075M KCl. After fixation in an acetic acid–alcohol mixture, they were centrifuged. This procedure was repeated twice. The pellet was resuspended in fixative and placed drop by drop on cold, damp slides. The slides were then dried and stained with a solution of Giemsa (diluted 1 : 50 in phosphate buffer, pH 6·8).

*Graftings.*—In order to check the condition of the cells, subcutaneous or intraocular grafts were regularly made (Markovits *et al.*, 1976). To do this, cells were trypsinized and then injected in a volume of 0·025 ml of PBS per animal. Newborn animals were used for the subcutaneous grafts, adults for the intraocular.

### RESULTS

### *Transplantability of untreated control cells*

Table I summarizes the data accumulated on the transplantability of untreated control cells.

Five lines from whole embryo, one line from foetal brain, and one from lung of young animals, were maintained *in vitro* for 1 to $2\frac{1}{2}$ years. During this long period of culture the different control lines behaved similarly. We could distinguish two periods: in the course of the first year, they generally kept their normal characteristics, and during the second year, underwent some changes which could constitute steps leading towards malignancy.

Table I shows that 6/7 control lines studied remained normal for the first 9–12 months (on average 17 population doublings) *in vitro*, and they did not form tumours in hamster, even after grafts of $2 \times 10^6$ cells per animal. In this first period, only one line (T59) originating from whole hamster embryo, gave rise to a tumour after 15 population doublings in hamster.

We have been able to maintain control cells in culture, sometimes up to 30 months (40 population doublings). During this second period (2nd and 3rd year of culture) control cells behaved similarly, whatever their origin: if a large number ($0{\cdot}5$–$2 \times 10^6$) of cells were grafted, tumours formed in some animals, but generally after a longer latency than usually observed for chemically transformed malignant cells. A graft of less than $0{\cdot}5 \times 10^6$ cells did not generally produce tumours (Table I).

### *Transplantability of whole embryo cells treated with non-carcinogenic compounds*

We have studied the transplantability of whole embryo cells treated with 9,12-dimethyl benzo(a)acridine and benzo(e)-

TABLE I.—*Transplantability of Control Hamster Cells in Hamster (Observations* 150–180 *Days after Grafting)*

| Cells | | | | | | |
|---|---|---|---|---|---|---|
| Origin | Code | Days in culture | Passages | Population doublings | No. cells inoculated per animal | No. tumour bearing animals/ No. animals receiving graft |
| Whole embryo* | T 14 | 350–600 | 16–26 | 16–26 | $1 \times 10^6$ | 0/6 |
| | | | | | $2 \times 10^6$ | 0/9 |
| | T 14 | 650–850 | 28–35 | 28–41 | $0 \cdot 1–1 \times 10^6$ | 0/17 |
| | | | | | $2 \times 10^6$ | 7/14 |
| | T 25 | 30–400 | 1–16 | 1–16 | $1–2 \times 10^6$ | 0/22 |
| | | 600 | 21 | 26 | $1 \times 10^6$ | 1/4 |
| | T 55 | 200–400 | 8–18 | 8–20 | $1 \cdot 3–2 \times 10^6$ | 0/10 |
| | T 58 | 260 | 10 | 10 | $2 \times 10^6$ | 0/3 |
| | | 380–410 | 16–18 | 17–20 | $0 \cdot 1–1 \cdot 3 \times 10^6$ | 0/18 |
| | | | | | $2 \times 10^6$ | 5/7 |
| | T 59 | 300 | 15 | 15 | $0 \cdot 1–0 \cdot 5 \times 10^6$ | 5/8 |
| | | | | | $2 \times 10^6$ | 9/10 |
| Foetal brain† | c-T | 210–300 | 7–9 | 7–10 | $0 \cdot 6 \times 10^6$ | 0/7 |
| | | 420 | 11 | 14 | $0 \cdot 5 \times 10^6$ | 6/6 |
| Baby hamster lung† | p-T | 580 | 20 | 21 | $1 \times 10^6$ | 0/10 |
| | | 750–860 | 23–29 | 24–33 | $1 \times 10^6$ | 1/13 |

* Subcutaneous grafting.

† Intraocular grafting.

pyrene, compounds which are not carcinogenic *in vivo*.

Table II shows that the transplantability of cells treated with noncarcinogenic compounds is similar to that of the untreated control cells. Cells treated with non-carcinogens are not transplantable during the first year and undergo changes from the second year of culture on.

The two compounds studied, which are not carcinogenic *in vivo*, do not cause transformation *in vitro* either. However, the non-carcinogenic chemicals were as toxic for the cells in culture as their carcinogenic isomers. We studied the toxic effects of the carcinogenic 7,9-dimethyl benzo(c)acridine and those of its non-carcinogenic isomer, 9,12-dimethyl benzo(a)acridine, and did not find any detectable difference between the toxic effects of the two isomers.

*Treatment of different hamster cells with carcinogenic compounds*

To compare the results of these experiments with those described in the preceding paragraphs, both the first appearance of malignancy and the degree of chemically induced malignancy must be considered.

(a) Hamster cells of different origins were treated with compounds which are carcinogenic *in vivo*: polycyclic hydrocarbons, a hetero-cyclic compound, and chlorobutadiene. All these carcinogenic chemicals induced malignant transforma-

TABLE II.—*Subcutaneous Transplantability of* 9,12-*Dimethyl Benz(a)acridine* (B.acr.neg.* 2 *μg/ml) and Benzo(e)pyrene* (B(e)P,* 0·1 *μg/ml) Treated Hamster Embryo Cells in Newborn Hamsters*†

| Cell line | Days in culture | Passage | Population doublings | No. cells inoculated per animal | No. tumour-bearing animals/ No. animals receiving graft |
|---|---|---|---|---|---|
| B. acr. neg. | 200 | 8 | 8 | $1 \times 10^5$ | 0/6 |
| | 350–400 | 14–17 | 14–17 | $1 \times 10^6$ | 1/7 |
| B(e)P | 30–250 | 2–11 | 2–11 | $0 \cdot 1–1 \cdot 2 \times 10^6$ | 0/16 |
| | 380–680 | 13–24 | 14–30 | $0 \cdot 3–2 \times 10^6$ | 5/22 |

* Non-carcinogenic *in vivo*.
† Time of observation after grafting = 150–180 days.

TABLE III.—*First Appearance of Malignancy in Chemically Treated Cells*

| Cells: Origin | Cells: Code | Days in culture | Passages | Population doublings | No. cells inoculated per animal | No. tumour-bearing animals/ No. animals receiving graft |
|---|---|---|---|---|---|---|
| Whole embryo* | B. acr. 2 | 135 | 6 | 6 | $0 \cdot 6 \times 10^6$ | 4/4 |
| | B(a)P 0·1 | 210 | 12 | 10 | $1 \times 10^6$ | 9/9 |
| | DMBA 0·05 | 270 | 18 | 18 | $2 \times 10^6$ | 9/9 |
| | BA 10 | 210 | 10 | 8 | $0 \cdot 6 \times 10^6$ | 6/6 |
| | BA 5 | 210 | 15 | 13 | $2 \times 10^6$ | 8/8 |
| | MCA 0·1 | 240 | 10 | 8 | $1 \cdot 5 \times 10^6$ | 4/6 |
| Foetal brain† | c-B(a)P 0·2 | 180 | 6 | 7 | $0 \cdot 6 \times 10^6$ | 3/3 |
| Baby hamster lung† | p-DMBA 0·05 | 600 | 22 | 27 | $1 \times 10^6$ | 1/4 |
| | p-CB 1 | 105 | 5 | 5 | $1 \times 10^6$ | 2/3 |

* Subcutaneous grafting.

† Intraocular grafting.

tion of the hamster cells (Markovits *et al.*, 1975*a*, 1976). In 7 cases, the malignancy manifested itself on average 6 months after treatment, corresponding to 8 population doublings (Table III).

After treatment with dimethylbenz(a)-anthracene (DMBA), we observed, in both whole embryo and baby lung cells, a relatively late appearance of malignancy (9 and 18 months; 18 and 27 population doublings respectively). Similar results were obtained in our recent experiments with DMBA-treated hamster brain cells. DMBA is a very potent carcinogen *in vivo*, but it is highly toxic *in vitro*. We could therefore use only a minimal concentration (0·05 $\mu$g/ml) of the compound. If this concentration is not optimal, that might be one of the reasons for the slow evolution of transformation process.

(b) Table IV shows the degree of malignancy of hamster cells transformed *in vitro* with chemical compounds. In order to determine the degree of malignancy, we grafted various numbers of these cells, and have thus been able to define for each line the minimum number required to produce tumours in a majority of the animals. Whole-embryo cells were grafted s.c., the other two types intraocularly.

Of the 6 transformed lines from whole embryo, 4 are highly malignant and produce tumours after grafts of 10 to 1000 cells, while the other two are weakly malignant and require grafts of at least 100,000 cells to produce tumours.

The lines originating from hamster lung and transformed by DMBA and 2-chlorobutadiene and from foetal brain cells transformed with benzo(a)pyrene are also

TABLE IV.—*Minimum Number of Chemically Transformed Hamster Cells Capable of Producing Tumours in Hamsters**

| Cells: Origin | Cells: Code | Passage | Days in culture | Minimum number of cells producing tumours |
|---|---|---|---|---|
| Whole embryo† | B. acr. | 19 | 300 | $1 \times 10^1$ |
| | B(a)P 0·1 | 29 | 550 | $5 \times 10^2$ |
| | DMBA 0·05 | 29 | 550 | $5 \times 10^2$ |
| | BA 10 | 27 | 530 | $1 \times 10^3$ |
| | BA 5 | 22 | 750 | $1 \times 10^5$ |
| | MCA 0·1 | 19 | 730 | $1 \times 10^5$ |
| Foetal brain‡ | c-B(a)P 0·2 | 21 | 480 | $1 \times 10^4$ |
| Baby hamster lung‡ | p-DMBA 0·05 | 29 | 780 | $1 \times 10^4$ |
| | p-CB 1 | 11 | 300 | $1 \times 10^4$ |

* Observation 150–180 days after grafting.
† Subcutaneous grafting.
‡ Intraocular grafting.

TABLE V.—*Characteristics of Normal and Chemically Treated Cells from Whole Hamster Embryos*

| Cells | Morphology | Saturation density cells/cm² | Ploidy | Colony formation in semi-solid medium (%) | Minimum number of cells producing tumours by s.c. grafting |
|---|---|---|---|---|---|
| Control | | | | | |
| Secondary | | $3 \times 10^4$ | Diploid | nil | No tumours |
| After 1 year *in vitro* | Normal | $6 \cdot 5 \times 10^4$ | Diploid | n.d. | |
| After 3 years *in vitro* | | $18 \times 10^4$ | Near-diploid | 0·05 | $>2 \times 10^6$ |
| Treated | | | | | |
| B(a)P (0·1 μg/ml)* | Transformed | $45 \times 10^4$ | Heteroploid | 5 | $5 \times 10^1$ |
| $BA_{10}$ (10 μg/ml)* | Transformed | $15 \times 10^4$ | Heteroploid | n.d. | $1 \times 10^3$ |
| MCA (0·1 μg/ml)* | Few signs of transformation | $8 \times 10^4$ | Near-diploid | n.d. | $1 \times 10^5$ |

n.d. = not done.

* = After 1½ years of culture.

malignant and produce tumours after grafts of 10,000 cells.

*Distinction between normal and transformed cells*

Normal cells are distinguished from cells transformed by chemical carcinogens not only by their neoplastic potential, but also by their morphology, growth characteristics, and karyotype (Table V).

After one year of culture, the properties of control cells from whole embryo were very similar to those of secondary cells:

FIG. 1.—Karyograph of T14-IR (cells from whole hamster embryo) after 2½ years of culture (distribution of modal number around diploid) and of cells transformed by 7-methyl benzo(a)anthracene (BA10-IR) after 1½ years of culture (distribution of modal number between diploid and tetraploid).

there was no observable morphological change; 93% of the cells remained diploid; they were not transplantable; their saturation density, however, doubled.

After 2½ years, the morphology of the control culture remained normal, but the saturation density tripled with respect to one-year-old cultures. The cells remained near-diploid (Fig. 1). When inoculated in semi-solid medium, only a small percentage of the cells formed three-dimensional colonies. At least $2 \times 10^6$ cells had to be grafted to produce tumours.

The whole-embryo cells transformed with benzo(a)pyrene (B(a)P) and 7-methyl benzo(a)anthracene (BA10) showed morphological changes: the cells became spindle-shaped, overlapped and criss-crossed. Their saturation density (especially of the B(a)P line) was very high. In addition, 80% of the cells of the B(a)P line are heteroploid. After 1½ years of culture, the B(a)P-transformed cells yielded 100 × more colonies in agar than the control cells after 2½ years. Grafts of 500 B(a)P-transformed cells produced tumours. As for the BA10-transformed cells, a large part of which are triploid (Fig. 1), 1000 cells were needed to produce tumours.

One of the properties of the BA10 cells is that they spontaneously form spheroids in suspension (Lévy *et al.*, 1976). These cells may therefore be an interesting model for radiobiological studies.

To obtain tumours from the 3-methyl-

TABLE VI.—*Characteristics of Normal and Chemically Treated Foetal Hamster Embryo Brain Cells*

| Cells | Morphology | Saturation density cells/cm$^2$ | Ploidy | Minimum number of cells producing tumours by intraocular grafting |
|---|---|---|---|---|
| Control line (after 9 months of culture) | Normal (glial) | $10 \times 10^4$ | Diploid | No tumours |
| B(a)P-treated (0·2 μg/ml) | Transformed | $28 \times 10^4$ | Hyperdiploid | $1 \times 10^4$ |

FIG. 2.—Karyograph of foetal hamster brain cells c-Tem after one year of culture (modal number clearly diploid) and of same cells transformed by benzo(a)pyrene: c-B(a)P 0·2 (modal number displaced toward hyperdiploid and tetraploid).

cholanthrene (MCA)-treated cultures, at least $10^5$ cells had to be injected per animal. Their morphology did not differ much from that of controls, their saturation density was not high and they remained near-diploid.

Table VI shows that there was also a correlation between the transplantability and certain *in vitro* properties of foetal hamster brain cells. After about 9 months in culture, control brain cells retained their normal morphology and karyotype (Fig. 2), and did not produce tumours. In contrast, B(a)P-transformed brain cells were morphologically very different, and had a saturation density almost 3 × higher. Their karyotype was slightly hyperploid (Fig. 2). They produced tumours when grafted in the hamster.

### DISCUSSION

It is now generally accepted that diploid mammalian cells have a finite *in vitro* lifetime. Hayflick and Moorhead (1961), and Hayflick (1965, 1975) suggest that this limited lifespan could not be related to the culture conditions but could reflect senescence at the cellular level. In commonly employed serum-supplemented media (such as the original or modified Eagle's medium), human fibroblasts die out after 50–70 cell generations.

Under the same conditions, Syrian hamster fibroblasts have a much shorter lifetime. Todaro and Green (1964), and Matsuya and Yamane (1970), using albumin-supplemented medium, were able to propagate diploid hamster embryo cells for about 30 cell generations (about 3 months).

We have obtained similar results in our experiments. We could maintain *in vitro*, in normal state, untreated hamster cells of various origins up to 26 cell generations (at least for 9–12 months), using a well buffered and enriched Eagle's medium (Markovits *et al.*, 1974). This medium enabled us to maintain low-passage hamster cells—both treated and untreated—which grow slowly. Se we could thus subculture them at a low rate: about once every 3–4 weeks. Under the same experimental conditions, chemically treated cells transformed and became malignant, whereas untreated cells kept their normal characteristics and remained nontransplantable. Thereby they provide good negative controls for our transformation experiments.

According to Huberman and Sachs (1966), toxic and transforming effects are two distinct properties of the chemical carcinogens. Our experiments seem to support this point of view. Sometimes the two properties are convergent: carcinogenic hydrocarbons such as B(a)P, which are very toxic, are also highly transforming *in vitro*, while others, such as the pollutant 2-chlorobutadiene (CB) are of low toxicity, but are highly carcinogenic (Markovits *et al.*, to be published).

The non-carcinogenic compounds are sometimes as toxic as their carcinogenic isomers, whereas cells treated with these non-carcinogenic compounds behaved like control cells.

### REFERENCES

BERWALD, Y. & SACHS, L. (1965) *In vitro* Transformation of Normal Cells to Tumor Cells by Carcinogenic Hydrocarbons. *J. natn. Cancer Inst.*, **35**, 641.

BOREK, C. (1976) *In vitro* Cell Transformation by Low Doses of X-irradiation and Neutrons. In *Biology of Radiation Carcinogenesis*. Eds J. M. Yuhas, R. W. Tennant and J. D. Regan. New York: Raven Press, p. 310.

DI PAOLO, J. A., DONOVAN, P. J. (1967) Properties of Syrian Hamster Cells Transformed in the Presence of Carcinogenic Hydrocarbons. *Exp. Cell Res.*, **48**, 361.

HAYFLICK, L. (1965) The Limited *in vitro* Lifetime of Human Diploid Cell Strains. *Exp. Cell Res.*, **37**, 614.

HAYFLICK, L. (1975) Current Theories of Biological Aging. *Fed. Proc.*, **34**, 9.

HAYFLICK, L. & MOORHEAD, P. S. (1961) The Serial Cultivation of Human Diploid Cell Strains. *Exp. Cell Res.*, **25**, 585.

HUBERMAN, E. & SACHS, L. (1966) Cell Susceptibility to Transformation and Cytotoxicity by the Carcinogenic Hydrocarbon Benzo(a)pyrene. *Proc. natn. Acad. Sci. U.S.A.*, **56**, 1123.

LEVY, S., PAPADOPOULO, D., NOCENTINI, S., CHAMAILLARD, L., BEESAU, O., HUBERT-HABART, M. & MARKOVITS, P. (1976) Formation and Growth of Spheroids in Culture of Hamster Embryo Cells Transformed by 7-methyl benz(a)anthracene (7-MBA). *Eur. J. Cancer*, **12**, 871.

MARKOVITS, P., COPPEY, J., PAPADOPOULO, D., MAZABRAUD, A. & HUBERT-HABART, M. (1974) Transformation Maligne de Cellules d'Embryon de Hamster en Culture par la diméthyl-7,10 benzo(c)-acridine. *Int. J. Cancer*, **14**, 215.

MARKOVITS, P. DAUDEL, P., PAPADOPOULO, D., MAZABRAUD, A. & HUBERT-HABART, M. (1975*a*) Sur la Cancérisation Chimique de Cellules d'Embryon de Hamster en Culture de Masse. *Bull. Cancer*, **62**, 59.

MARKOVITS, P., PAPADOPOULO, D. HUBERT-HABART, M., MANN, S. K., LABREQUE, A. & SABHARWAL, P. S. (1975*b*) Establishment of Normal Diploid and Malignant Heteroploid Cell Lines from Non-treated and Benzo(a)pyrene treated Hamster Embryo Cell Cultures. *Experientia*, **31**, 1215.

MARKOVITS, P., LÉVY, S., NOCENTINI, S., MAZABRAUD, A., VELIZAROF, A., SABHARWAL, P. S. & BENDA, P. (1976) Transformation Maligne *in vitro* de Cellules de Cerveau Foetal de Hamster par le Benzo(a)pyrene. *C.R. Acad. Sci.*, **282**, 2015.

MATSUYA, Y. & YAMANE, J. (1970) Establishment of Hamster Fibroblast Culture in Albumin Fortified medium. *Proc. Soc. exp. Biol. Med.*, **135**, 393.

TODARO, G. & GREEN, H. (1964) Serum Albumin Supplemented Medium for Long Term Cultivation of Mammalian Fibroblast Strains. *Proc. Soc. exp. Biol. Med.*, **116**, 688.

YERGANIAN, G. & LAVAPPA, K. (1971) Procedures for Culturing Diploid Cells and Preparation of Meiotic Chromosomes from Dwarf Species of Hamsters. In *Chemical Mutagens*. New York–London: Plenum Press. **2**, p. 387.